# Labellum structure of *Bulbophyllum echinolabium* J.J. Sm. (section *Lepidorhiza* Schltr., Bulbophyllinae Schltr., Orchidaceae Juss.)

**DOI:** 10.1007/s00709-019-01372-4

**Published:** 2019-04-16

**Authors:** Natalia Wiśniewska, Monika M. Lipińska, Marek Gołębiowski, Agnieszka K. Kowalkowska

**Affiliations:** 1 Department of Plant Cytology and Embryology, Faculty of Biology, University of Gdańsk, Wita Stwosza 59, 80-308 Gdańsk, Poland; 2 Department of Plant Taxonomy and Nature Conservation, Faculty of Biology, University of Gdańsk, Wita Stwosza 59, 80-308 Gdańsk, Poland; 3 Laboratory of Analysis of Natural Compounds, Department of Environmental Analytics, Faculty of Chemistry, University of Gdańsk, Wita Stwosza 63, 80-952 Gdańsk, Poland

**Keywords:** Histochemistry, Micromorphology, Ultrastructure, GC/MS, Sapromyophily, Fly-pollination syndrome

## Abstract

This micromorphological, chemical and ultrastructural study is a continuation of research conducted on the section *Lepidorhiza*. The *Bulbophyllum echinolabium* flowers comprised features that characterize a sapromyophilous syndrome, having large, glistening parts that emit an intense scent of rotten meat. The secretory activity was described in the hypochile (nectary in longitudinal groove and in the prickles) and the epichile (putative osmophore). The ultrastructural studies revealed a dense cytoplasm in the epidermis and subepidermal tissue with large nuclei and numerous mitochondria, the profiles of SER and RER, and dictyosomes. Large amounts of heterogeneous residues of secreted material (possibly phenolic) were present on the cuticle surface, similar to the unusual prominent periplasmic space with flocculent secretory material. The chemical analysis (GC/MS) of the scent profile of lips comprised carbohydrates and their derivatives (29.55% of all compounds), amino acids (1.66%), lipids (8.04%) and other organic compounds (60.73%). A great number of identified compounds are Diptera attractants (mainly Milichiidae, Tephritidae, Drosophilidae, Muscidae, Sarcophagidae, Tachinidae). The examination of visual and olfactory features indicates correlation between colour of flowers and the type of olfactory mimicry, where a dark colour labellum emits strong smell of rotten waste.

Floral scent, colour and texture are important attractants for pollinators, such as flies, imitating their brood and feeding sites (Dobson 2006). The strong smell in sapromyophilous flowers acts as a long-distance attractant and helps flies find the flowers hidden in vegetation. Colour and texture interplay with the floral scent (Jürgens et al. 2006). Calliphoridae and Sarcophagidae flies prefer yellow flowers with the presence of a sweet odour, which signals their food sources, or brown-purple colour in combination with an excrement odour, which signals egg-laying sites (Dobson 2006). Specialized devices, such as motile elements of the perianth, support pollination accuracy and effectiveness (Christensen 1994; Borba and Semir 1998; Vogel 2001; Kowalkowska et al. 2015a). The fly-pollinated orchids (myophilous or sapromyophilous) are described in the genus *Bulbophyllum* Thou. *Bulbophyllum lasianthum* Lindl. (section *Beccariana* Pfitz.), *Bulbophyllum lobbii* Lindl. (section *Sestochilos* Benth & Hook.f.) and *Bulbophyllum virescens* J.J. Sm. (section *Beccariana*) (Ong and Tan 2011) are pollinated by blow flies (Calliphoridae). Flesh flies (Sarcophagidae) pollinate *Bulbophyllum mandibulare* Rchb.f. (section *Lepidorhiza* Schltr.) (Ong and Tan 2012), *Bulbophyllum subumbellatum* Ridl. and *B. virescens* (Ong and Tan 2011), both from section *Beccariana*. Whereas fruit flies, such as *Bactrocera* spp., are reported from *Bulbophyllum baileyi* F. Muell. (section *Sestochilos*) (Tan and Nishida 2007).

*Bulbophyllum* sect. *Lepidorhiza* comprises 28 species, with Borneo, Sulawesi, the Philippines and New Guinea as the main centres of diversity, growing in lowland or montane forests at elevations up to 1900 m a.s.l. (Pridgeon et al. 2014). In representatives of this section, the secretory tissue is located superficially in the well-defined, longitudinal, median lip groove, which is a highly conservative lip feature among *Bulbophyllum*, in contrast to the nature of the secretion (Teixeira et al. 2004; Nunes et al. 2014; Kowalkowska et al. 2015a; Wiśniewska et al. 2018). In *Bulbophyllum* representatives, groove secretion has been identified as nectar, e.g. *Bulbophyllum epiphytum* (section *Micranthae* Barb. Rodr.), *Bulbophyllum glutinosum* (section *Napellii* Rchb.f.), *Bulbophyllum regnellii* (section *Napellii*), *Bulbophyllum rothschildianum* (section *Cirrhopetalum* Lindl.) (Teixeira et al. 2004) and *Bulbophyllum wendlandianum* (section *Cirrhopetalum* Lindl. or *Cirrhopetaloides* Garay, Hamer & Siegerist) (Kowalkowska et al. 2015a), a protein-rich mucilaginous secretion in the section *Racemosae* (Davies and Stpiczyńska 2014) or a mucilaginous secretion in *Bulbophyllum weberi* (section *Cirrhopetalum*) (Kowalkowska et al. 2017). The scent glands (osmophores) occur on the apices of tepals, as the swollen tips of tepal appendages or as unicellular trichomes on labellar lobes (i.e. Teixeira et al. 2004; Kowalkowska et al. 2015b). In the previously examined *Bulbophyllum levanae* and *Bulbophyllum nymphopolitanum* from the section *Lepidorhiza* (Wiśniewska et al. 2018), the putative osmophores, based on phenols detected in the plastoglobuli of plastids, were located on the extended apices of sepals and possibly on petals. An abundance of proteins detected in the labellum of both species is most likely associated with the unpleasant scent of the flowers, whereas the lipid-rich cuticular striations formed a thin wax layer on the epidermis which is presumably involved in the brilliance of floral tepals, strongly attracting flies (Kugler 1951, apud Meve and Liede 1994). The presence of predominant periplasmic spaces in secretory cells with heterogeneous material gathered beneath the cuticle occurs in the labellum epidermis in these two species (Wiśniewska et al. 2018).

*Bulbophyllum echinolabium* J.J. Sm. (section *Lepidorhiza*) is endemic to the Indonesian island of Sulawesi, where it appears to be restricted to a single range of hills (Wood 2005). It is known for its flowers, which reach up to 35 cm long (possibly the largest flowers in the genus) and are borne from an inflorescence that may attain 70 cm in height. The labellum is reddish with a dark purple, thick, fleshy, carunculate-echinate hypochile and a smooth epichile, which is canaliculate with revolute margins (Wood 2005). The flowers of *B. echinolabium* imitate a highly unpleasant smell of rotten waste, which is characteristic of sapromyophilous flowers.

The proposed studies are the continuation of an anatomical survey of species from the section *Lepidorhiza*. The aims of the present work were (a) to verify the presence of secretion in labellum; (b) to examine in detail the secretory tissue; (c) to compare the anatomical similarities and differences in the micromorphology, anatomy and ultrastructure of secretory tissue of *B. echinolabium* with previously published *B. levanae* and *B. nymphopolitanum* (*Lepidorhiza*) (Wiśniewska et al. 2018); and (d) to characterize the floral scent profile and compare results in order to verify if there is a correlation between colour of flowers and the type of olfactory mimicry. This is the first anatomical study of the secretory tissue of *B. echinolabium.* Provided studies will give more insight into understanding fly-pollination syndrome.

## Materials and methods

Tissue samples were collected from fresh flowers at anthesis from the greenhouse at the University of Gdańsk, Faculty of Biology (voucher numbers: AKK 2016-001, ML 2375).

To fix plant material 2.5% (*v*/*v*), glutaraldehyde (GA) in 0.05 M cacodylate buffer (pH = 7.0) was used. Material for light microscopy (LM) following fixation was rinsed with cacodylate buffer and then dehydrated in an ethanol series. Then, the material was embedded in methylmethacrylate-based resin (Technovit 7100, Heraeus Kulzer GmbH). Sections were cut with glass knives (5–7 μm thick) using a Sorvall MT 2B and a Leica EM UC 7 ultramicrotomes and mounted on glass slides. Transverse sections were presented from different portions (apical, middle and basal in epichile, groove and keel, prickles on the hypochile).

For histochemical analysis, semi-thin control sections for light microscopy were stained with 0.05% (*w*/*v*) aqueous Toluidine Blue O (TBO, C.I. 52040) (Feder and O’Brien 1968; Ruzin 1999). Aniline Blue Black (ABB, C.I. 20470) was used for the detection of water-insoluble proteins (Jensen 1962). The Periodic Acid-Schiff reaction (PAS) was used to identify the presence of water-insoluble polysaccharides (Jensen 1962). A 0.05% (*w*/*v*) aqueous Ruthenium Red (C.I. 77800) solution and a 10% (*w*/*v*) aqueous solution of FeCl_3_ were used to test for pectic acids/mucilage (Johansen 1940) and catechol-type dihydroxyphenols (Gahan 1984), respectively. The preparations were examined and photographed with a Nikon Eclipse E 800 light microscope and a Nikon DS-5 Mc camera using Lucia Image software (University of Gdańsk, Poland). The sections, following FeCl_3_ staining, were observed using the differential interference contrast (DIC) imaging. Auramine O (C.I. 41000) 0.01% (*w*/*v*) solution in 0.05 M buffer Tris/HCl, pH = 7.2 was used to detect the presence of cuticle (Heslop-Harrison 1977), especially unsaturated acidic waxes and cutin precursors (Gahan 1984). Nucleus structure was examined in preparations stained with the fluorochrome 4′,6-diamidino-2-phenylindole (DAPI). Staining reactions with Auramine O and DAPI were examined with a Nikon Eclipse E800 fluorescence microscope equipped with filter B-2A (EX 450–490 nm, DM 505 nm, BA 520 nm).

Following dehydration in an ethanol series, the samples were prepared and subjected to critical-point drying using liquid CO_2_, coated with gold and examined for micromorphological studies by scanning electron microscopy (SEM) using a Philips XL-30 at an accelerating voltage of 15–20 kV (Laboratory of Electron Microscopy, University of Gdańsk, Poland).

To analyse ultrastructural features in transmission electron microscopy (TEM), labellum was fixed in 2.5% (*v*/*v*) glutaraldehyde (GA) in 0.05 M cacodylate buffer (pH 7.0). The material was then post-fixed overnight in 1% (*w*/*v*) OsO_4_ in cacodylate buffer in a refrigerator and finally rinsed in buffer. After 1 h in a 1% (*w*/*v*) aqueous solution of uranyl acetate, the material was dehydrated with acetone and embedded in Spurr’s resin (Spurr 1969). Semi-thin sections (0.8–1 μm thick) were mounted on glass slides and treated with a 0.3% (*w*/*v*) ethanolic solution of Sudan Black B (SBB, C.I. 26150) for lipid localization (Bronner 1975), observed with a Nikon Eclipse E800 light microscope. Ultrathin sections were cut on a Leica EM UC7 ultramicrotome with a diamond knife and stained with uranyl acetate and lead citrate (Reynolds 1963). Samples were prepared for the above mentioned observations in accordance with previously described procedures (i.e. Kowalkowska et al. 2010; Krawczyk et al. 2016; Święczkowska and Kowalkowska 2015; Naczk et al. 2018). The sections were examined in a Tecnai G2 Spirit BioTwin FEI transmission electron microscope (Laboratory of Electron Microscopy, University of Gdańsk, Poland) at an accelerating voltage of 120 kV.

To identify the chemical composition of lips, they were extracted in 20 ml of dichloromethane and methanol (separately) for 15 min. The extracts were filtered and collected in a glass vial. Solvents were evaporated under nitrogen to 4 ml at room temperature and extracts were kept at 4 °C until analysed. Sugars were identified as native compounds and as trimethylsilyl derivatives (TMSi). Silylized derivatives were obtained by the addition of 0.1 ml BSTFA + TMCS (99:1; Sigma Aldrich) to 1 mg of each extract, and left for 1 h at 100 °C (Evershed 1992; Christie 1994). The samples were analysed by gas chromatography/mass spectrometry on a GC/MS QP-2010 SE (Simadzu, Kyoto, Japan) equipped with a fused silica capillary column ZB-5, 30 m × 0.25 mm i.d., and with a film 0.25 μm thick. Helium was used as the carrier gas at a flow rate of 1 ml min^−1^. Electron impact mode was performed at 70 eV. The injector and GC-MS interface temperatures were held at 310 °C. The ion source was maintained at 200 °C. The split ratio was 1:20, and the injection volume was 1 μL. The column temperature was programmed from 40 to 310 °C at 4 °C min^−1^ and then held at 310 °C for 10 min. Organic compounds were identified on the basis of characteristic silyl derivative ions or on the basis of the mass spectra obtained for native compounds. The relative composition of the fraction was determined based on the peak areas from the total ion current (TIC).

## Results

**Fig. 1:**
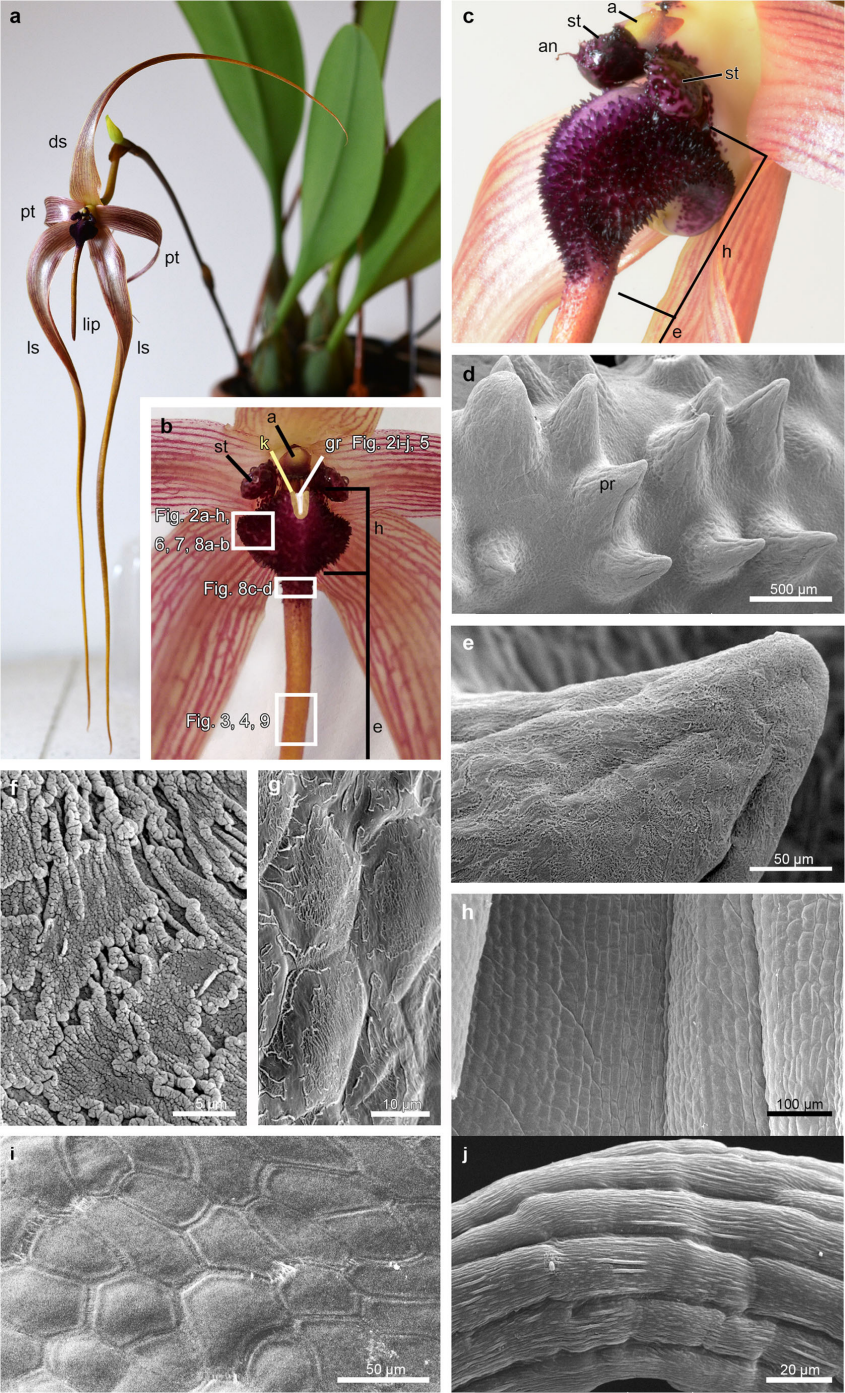
Macro and micromorphological features of flowers of *Bulbophyllum echinolabium*. **a** Flower. **b**, **c** Details of the flower. **d** The adaxial (inner) surface of the hypochile composed by prickles. **e** Detail of **d**, the prickle. **f**, **g** Fragmented cuticle on the adaxial surface of the epidermis: apical (**f**) and basal (**g**) part of prickle. **h** Smooth cells on the abaxial (outer) surface of the epichile. **i** Inner (adaxial) smooth surface of the stelidia. **j** Detail of the appendage of the stelidium with elongated cells. *a* anther-cap, *an* appendage of stelidium, *ds* dorsal sepal, *e* epichile, *g* gynostemium, *gr* groove (marked with white on **b**), *h* hypochile, *k* keel (marked with yellow on **b**), *ls* lateral sepal, *pr* prickle, *pt* petal, *st* stelidium

**Fig. 2:**
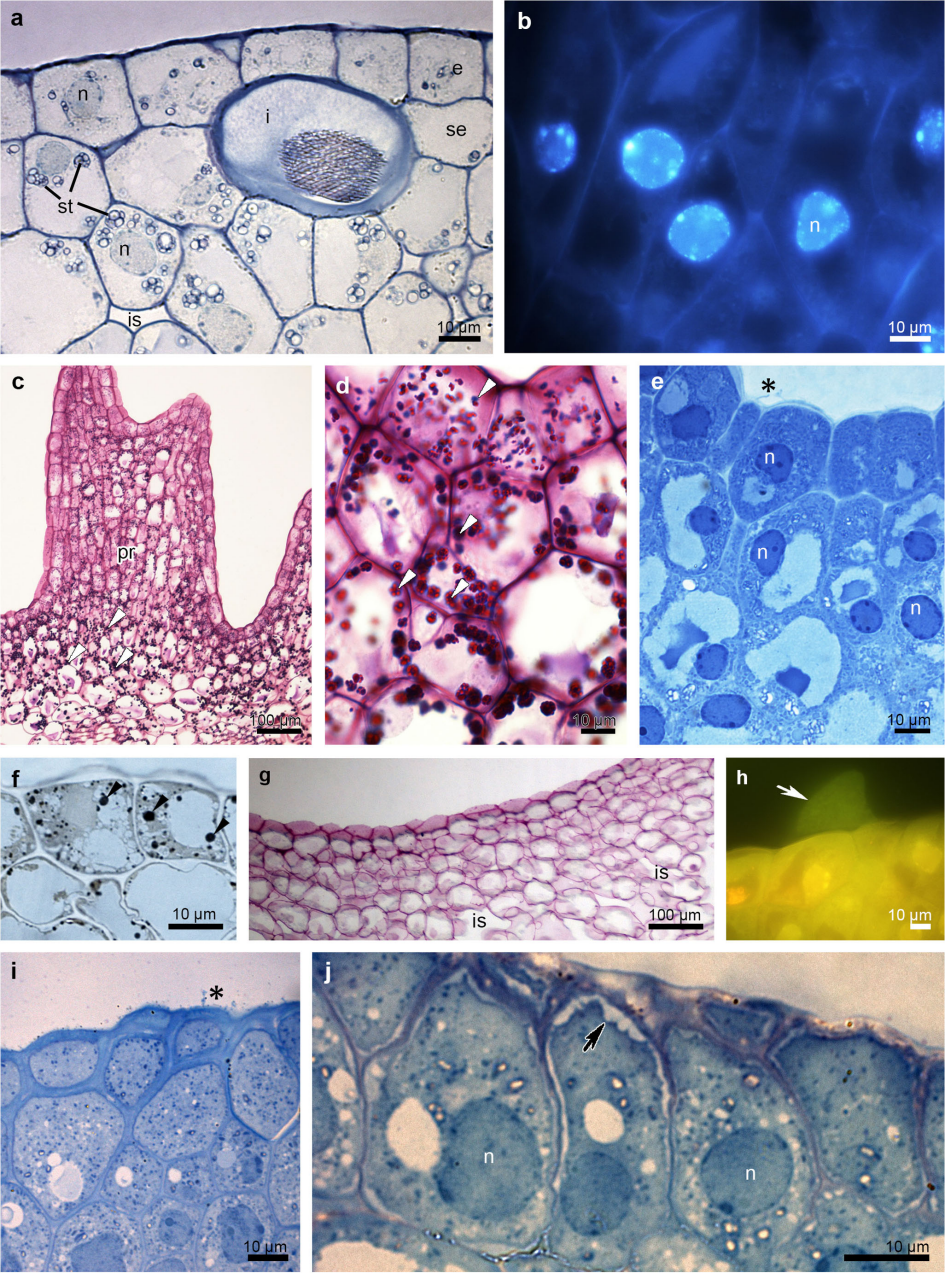
Histochemical features of the adaxial surface of the hypohile: the prickles (**a–h**) and the groove (**i**, **j**) of *B. echinolabium.***a** Transverse section showing a single-layered epidermis and subepidermal cells and idioblasts with raphides (TBO). **b** Subepidermal cells with enlarged nuclei (DAPI). **c** Abundance of starch grains (*white arrowheads*, PAS). **d** Detail of **c**. **e** The epidermis and subepidermal tissue stained intensively for proteins (ABB), note the secretory material on the surface (*black asterisk*). **f** Lipid drops (*black arrowheads*) (SBB). **g** Staining with ruthenium red with no mucilage. **h** Wax-like material on the surface of the epidermis (*white arrow*, Auramine O). **i** Epidermis and subepidermal secretory tissue of the groove, note the secretory material on the surface (*black asterisk*) (TBO). **j** Details of the groove epidermis with periplasmic spaces beneath the cuticle (*black arrow*) (TBO). *e* epidermis, *i* idioblast, *is* intercellular spaces, *n* nuclei, *se* subepidermal tissue, *st* starch, *vb* vascular bundle

Living flowers of *B. echinolabium* (Fig. 1a) were approximately 25 cm long with glistening, yellowish with lilac-pink nerves tepals (Fig. 1a, b), a pale yellow column with dark purple stelidia and a yellow with purple anther-cap (Fig. 1b, c). In the labellum, attached to the base of the floral column by a small, springy hinge, two macromorphologically different areas were distinguished: the reddish hypochile and the yellowish, canaliculate epichile (Fig. 1b). The fleshy and echinate hypochile had a brighter groove between keels (Fig. 1b, c). The prickles (according to Wood 2005) were multicellular outgrowths (Figs. 1d, e and 2c), covered with fragmented cuticle (Fig. 1f, g, compare with Fig. 5a, c, d). In contrast, the adaxial surface of epichile was built by smooth cells, rectangular in shape, and without any cuticle striations or residues on the outer surface (Fig. 1h). Likewise, epidermal cells of stelidia were polygonal and smooth on the inner surface (Fig. 1i) and elongated and with slight cuticular striations on the appendage (Fig. 1j), which did not indicate secretion.

**Fig. 3:**
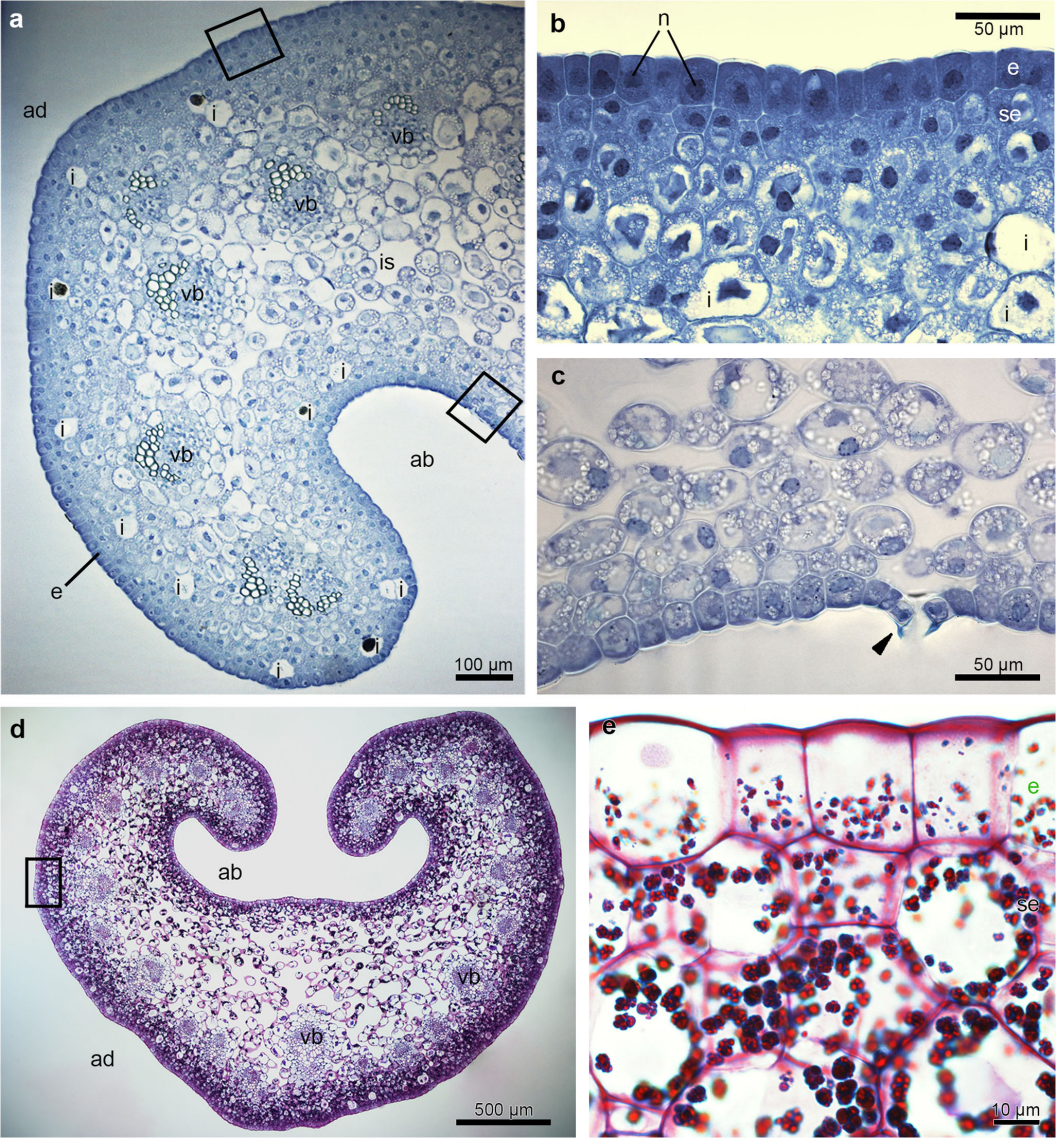
Histochemical features of the *B. echinolabium* epichile (transverse sections)*.***a** General view showing a single layer of epidermis, a few layers of subepidermal cells with idioblasts and several collateral vascular bundles in the ground parenchyma (TBO). **b** Detail of transverse section (**a**) (*square*) epidermis with dense cytoplasm and enlarged nuclei stained intensively for proteins (ABB). **c** Detail of **a** (*square*), abaxial surface of the epichile with stoma (*arrowhead*, TBO). **d** Transverse section of the epichile tissue (PAS). **e** Details of **d** (*square*), abundance of starch grains in subepidermal tissue. *ab* abaxial surface, *ad* adaxial surface, *e* epidermis, *i* idioblast, *is* intercellular spaces, *n* nuclei, *se* subepidermal tissue, *vb* vascular bundle

**Fig. 4:**
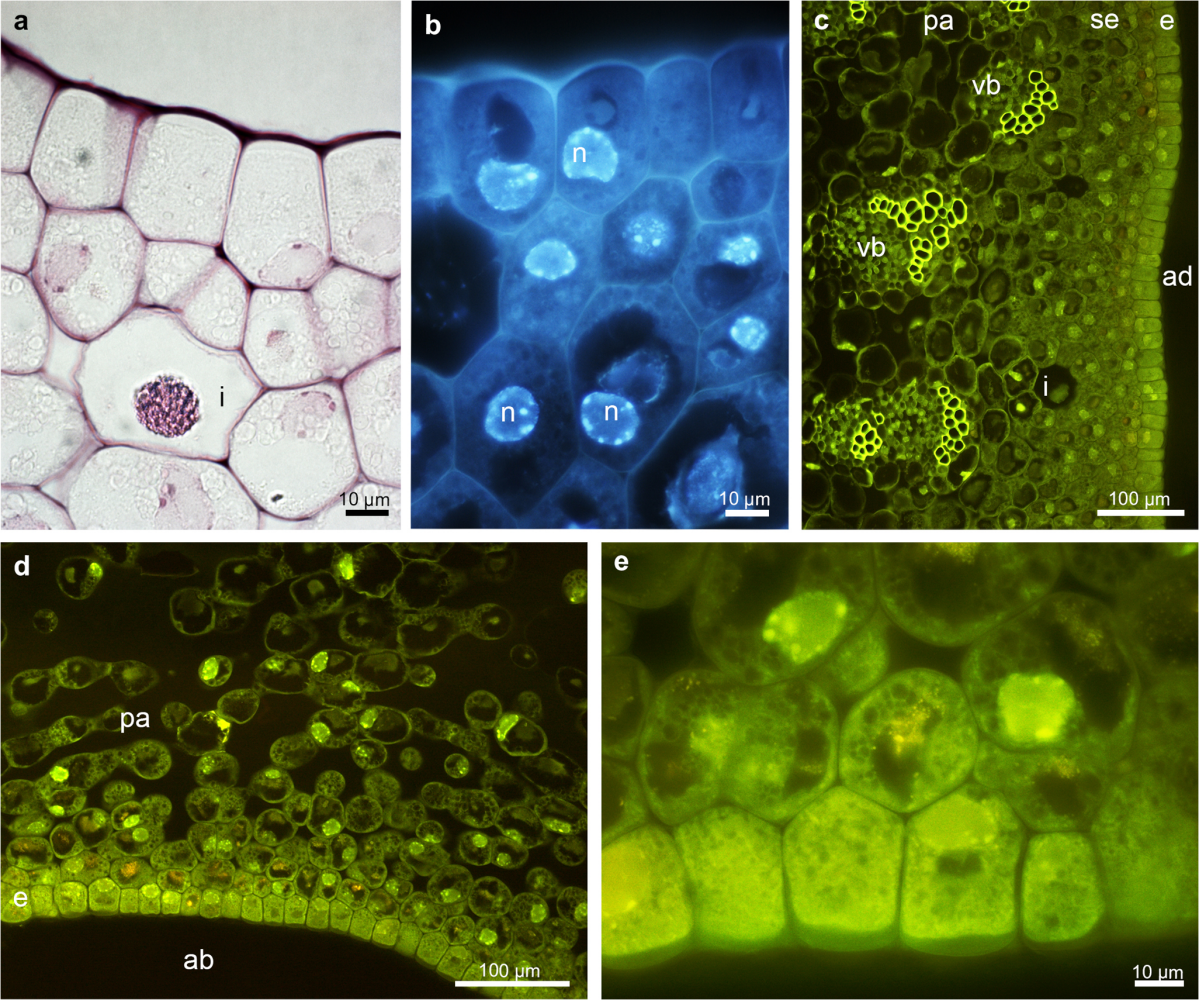
Histochemical features of the middle part of the epichile of *B. echinolabium.***a** Transverse section: the epidermis and few layers of subepidermal cells with idioblasts following staining with ruthenium red. **b** The epidermis and subepidermal tissue with enlarged, nuclei (DAPI). **c**, **d** Transverse sections of adaxial (inner) and abaxial (outer) surfaces stained with Auramine O. **e** Detail of **d**. *ab* abaxial surface, *ad* adaxial surface, *e* epidermis, *i* idioblast, *n* nuclei, *pa* parenchyma, *vb* vascular bundle

There were no significant differences at a histochemical level between particular parts (groove and prickles of hypochile, apical and basal parts of epichile), therefore they will be described in general way as hypochile and epichile. In the transverse section, the adaxial part of both hypochile and epichile contained a single-layered epidermis, few layers of subepidermal cells and a deeply located ground parenchyma consisting of larger, more vacuolated cells, through which numerous collateral vascular bundles ran (Figs. 2c and 3a, d). Large idioblasts comprising raphides were also present in the adaxial subepidermal cells of hypochile (Fig. 2a) and epichile (Fig. 3b). The subepidermal parenchyma comprised a few layers of cells containing dense parietal cytoplasm with enlarged nuclei (stained with DAPI, Figs. 2b, 4b). In both prickles and the groove, the secretory material was noted on the surface (Fig. 2e, i). Only the epidermal cells of the groove exhibited periplasmic spaces beneath the cuticle (Fig. 2j). Histochemical results also showed a great number of similarities between the tissue of hypochile and epichile. An abundance of proteins was observed in the adaxial epidermis and subepidermal tissue, although there was a distinctly greater amount in the epichile (Figs. 2e and 3b). The stomata were present on the abaxial surface of the epichile (Fig. 3c). Starch occurred throughout the labellum, but was most profusely found in adaxial subepidermal tissue of both hypochile (Fig. 2c, d) and epichile (Fig. 3d, e). Single lipid droplets were detected mostly in the epidermis and subepidermal cells (Fig. 2f). Additionally, the test with ruthenium red did not reveal the presence of mucilage neither in hypochile (Fig. 2g) or epichile (Fig. 4a). The unsaturated acidic waxes were detected in the cuticle (Auramine O, Figs. 2h and 4c–e).

**Fig. 5:**
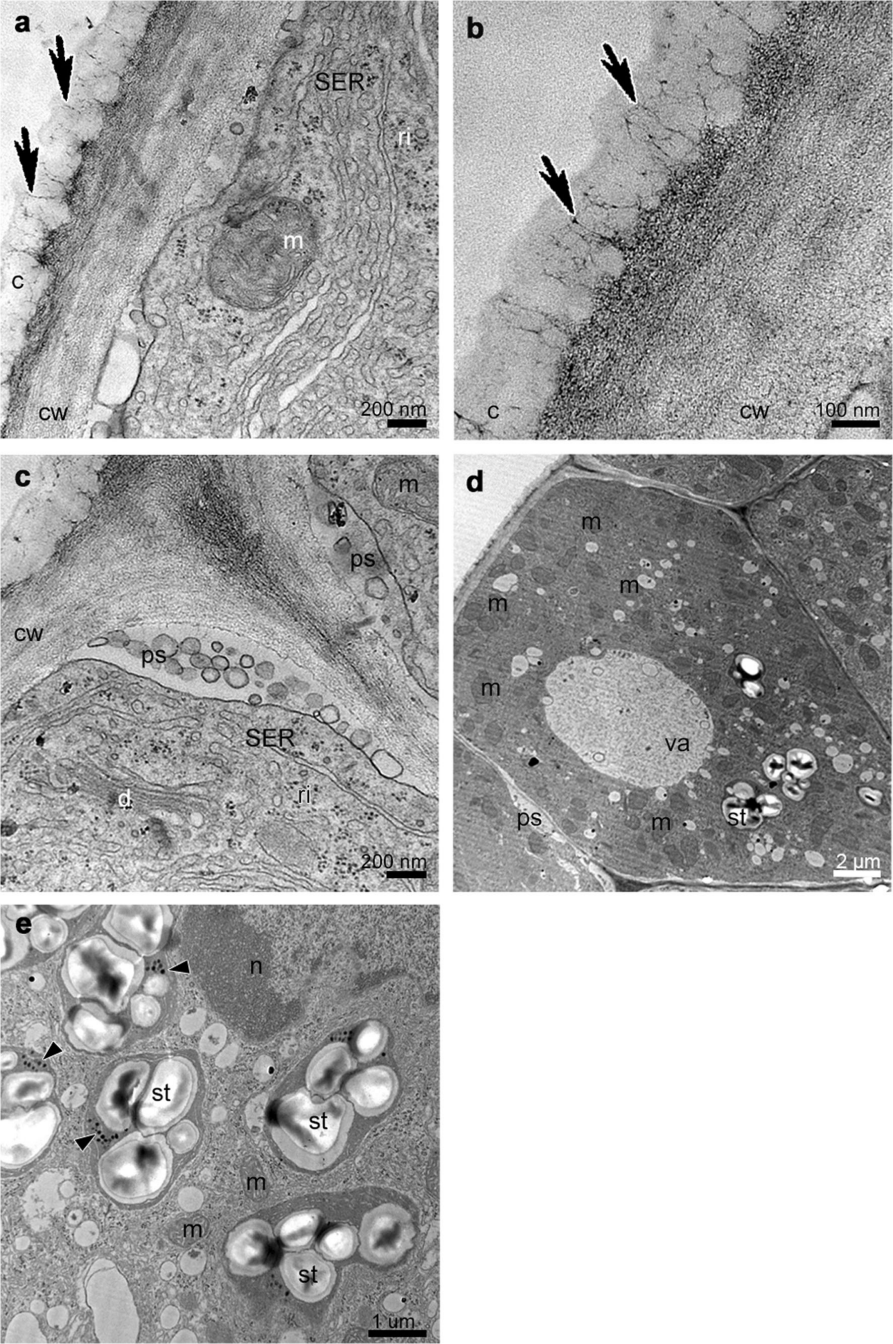
Ultrastructural analysis (TEM) of the groove of the hypochile showing secretory epidermal cells with heterogeneous cell wall and micro-channels in cuticle (*black arrows*, **a**, **b**), periplasmic space with flocculated secretory material and numerous vesicles building into plasmalemma (**a**, **c**), profiles of SER (**a**, **c**), fully developed dictyosomes (**c**), mitochondria (**d**), ribosomes (**d**), plastids with starch grains (**d**, **e**) and plastoglobuli (*black arrowheads*, **e**). *c* cuticle, *cw* cell wall, *d* dictyosome, *m* mitochondrion, *n* nucleus, *ps* periplasmic space, *ri* ribosomes, *st* starch grains, *va* vacuole

**Fig. 6:**
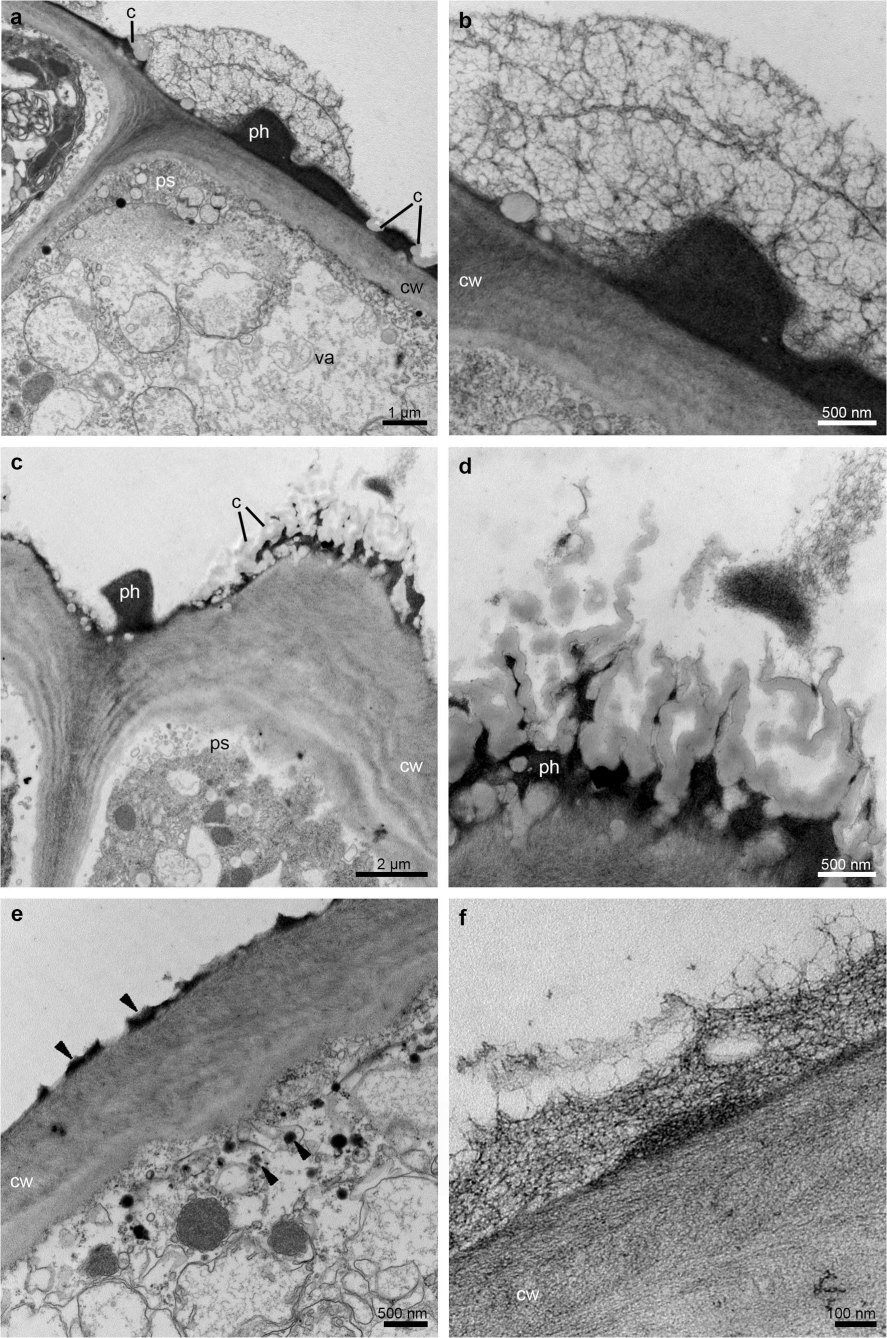
Ultrastructural analysis (TEM) of the appendix of the prickle in the middle part of the hypochile. **a** Sections through the epidermal cell wall with periplasmic spaces beneath and large amounts of heterogeneous residues of secreted material with fragmented pieces of the cuticle on the surface. **b** Detail of **a**. **c** Phenolic secretory material on the cuticle surface and periplasmic spaces beneath the cell wall. **d** Detail of **c**. **e** Phenolic material on the cuticle surface (*black arrowheads*) and inside the numerous vesicles fusing with the plasmalemma. **f** Detail of the epidermis cell wall with secretory material on the surface. *c* cuticle, *cw* cell wall, *ph* phenolic secretion, *ps* periplasmic space, *va* vacuole

**Fig. 7:**
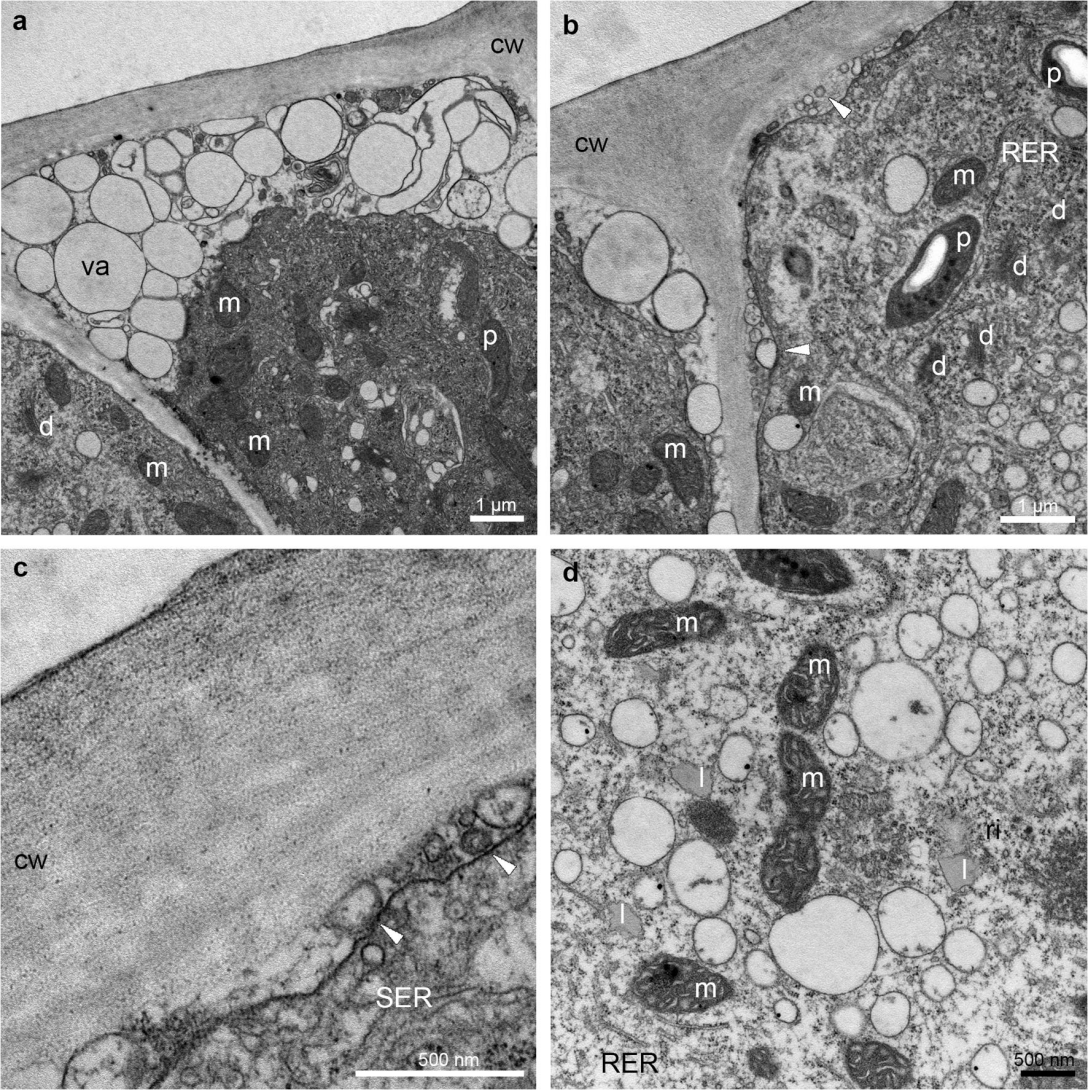
Ultrastructural details (TEM) of the prickle (hypochile) showing secretory epidermal cells with dense cytoplasm, numerous small vacuoles in close vicinity of the cell wall (**a**, **b**), an abundance of mitochondria, plastids with starch grains and plastoglobuli (**a**, **b**, **d**), fully developed dictyosomes (**a**, **b**), profiles of smooth (SER) and rough endoplasmic reticulum (RER) (**b–d**), lipid droplets (**d**) and periplasmic space with flocculated secretory material and numerous vesicles building into plasmalemma (*white arrowheads*) (**b**, **c**). *cw* cell wall, *d* dictyosome, *l* lipid droplet, *m* mitochondrion, *p* plastid, *ps* periplasmic space, *va* vacuole

**Fig. 8:**
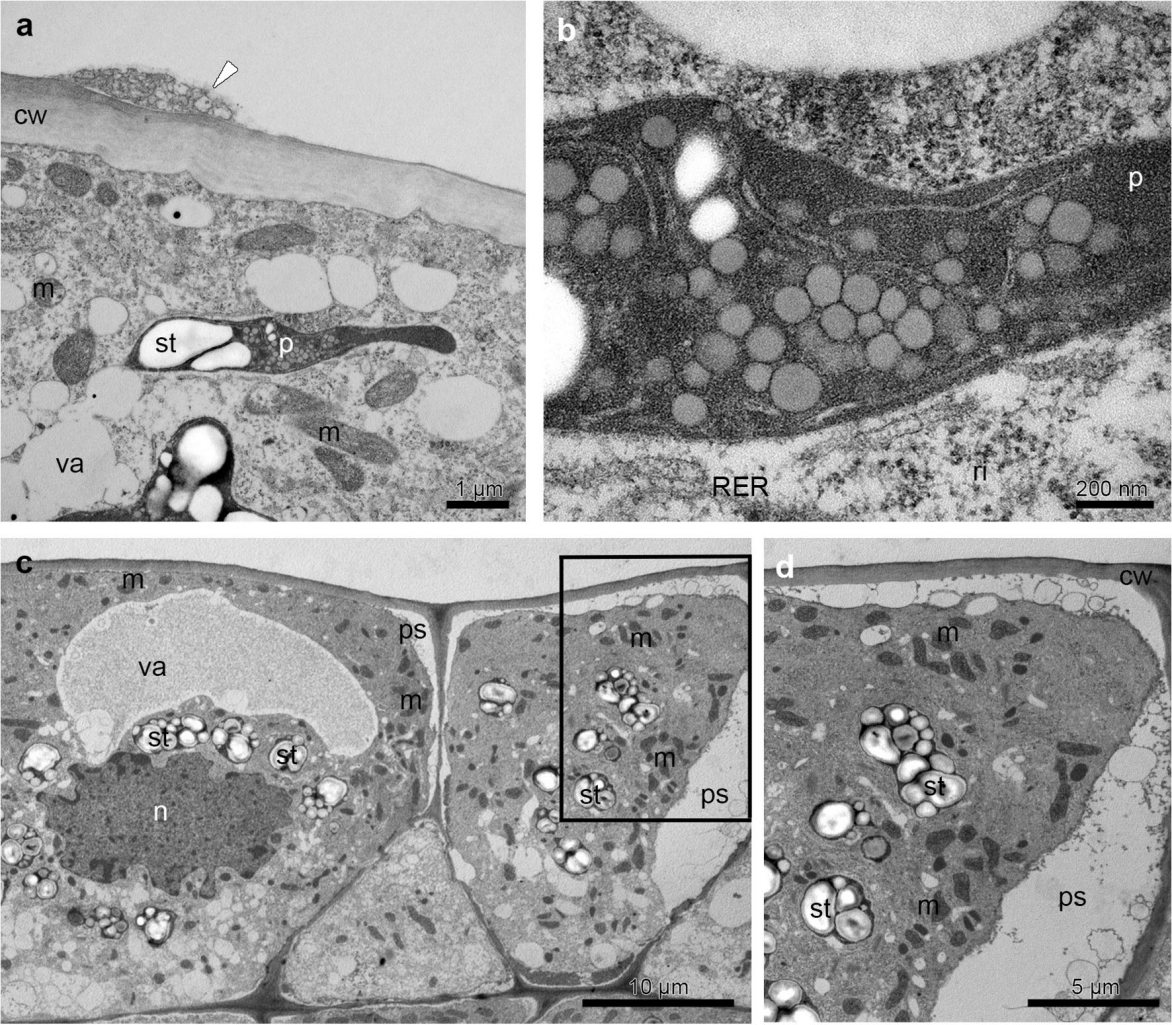
Ultrastructural analysis (TEM) of the prickles on the hypochile (**a**, **b**) and basal part of the epichile (**c**, **d**) revealed **a** residues of secretory material on the cuticle surface (*white arrowhead*), plastids with plastoglobuli, tubules and starch grains (**a–d**) and profiles of SER and RER (**b**), numerous mitochondria (**a–d**). **c** Epidermal cells containing a prominent periplasmic space with flocculent material (**c**, **d**, **d** detail of **c**), dense cytoplasm with enlarged nuclei (**c**). *cw* cell wall, *d* dictyosome, *m* mitochondrion, *n* nuclei, *p* plastid, *ps* periplasmic space, *ri* ribosomes, *st* starch grains, *va* vacuole

**Fig. 9:**
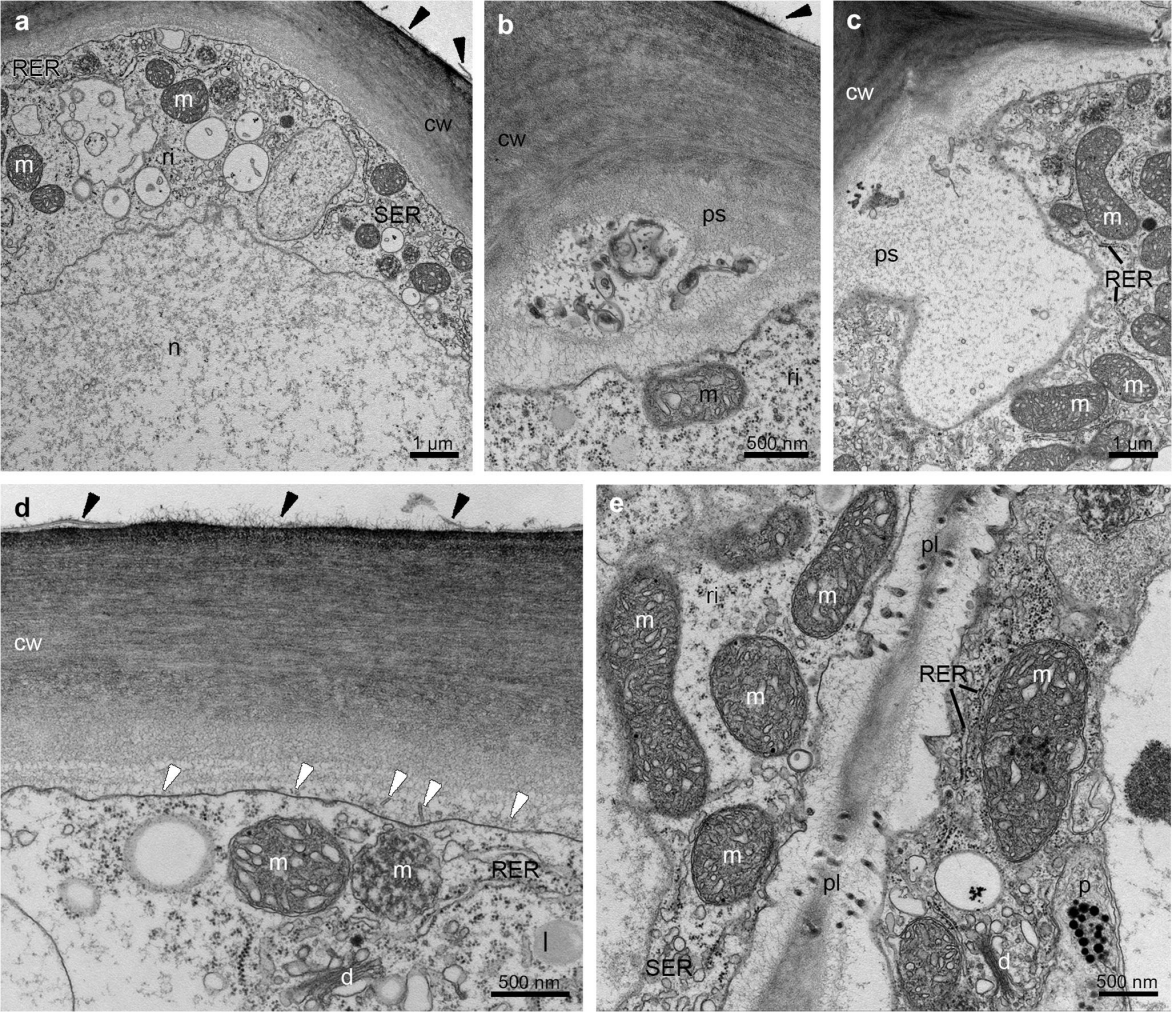
Ultrastructural details (TEM) of the adaxial surface of the middle part of the epichile revealed epidermal cells with secretory material (*black arrowheads*) (**a**, **b**, **d**), dense cytoplasm with numerous mitochondria (**a–e**), prominent periplasmic space with flocculent material (**b**, **c**), abundant SER and RER (**c–e**), fully developed dictyosomes (**d**, **e**), plasmalemma with irregular outline and vesicles building into it (*white arrowheads*) (**d**), plasmodesmata connecting adjacent epidermal cells (**e**) and secretory material on surface (**d**), lipid bodies (**d**), plastids-chromoplasts with plastoglobuli (**e**). *cw* cell wall, *d* dictyosome, *l* lipid bodies, *m* mitochondrion, *n* nuclei, *p* plastid, *pl* plasmodesmata, *ps* periplasmic space, *ri* ribosomes

The TEM studies have been made on groove (Fig. 5) and prickles (Figs. 6, 7 and 8a, b) (hypochile) and adaxial part of the epichile (Figs. 8c, d and 9). The TEM examination revealed no secretory material on the epidermal cells of groove (Fig. 5a, b) in contrast to large amounts of heterogeneous, possibly phenolic residues of secreted material on the cuticle surface of the prickle cells (Figs. 6a, b, d, f and 7a, b, d). Likewise, in the adaxial surface of the epichile, epidermal cells with small amounts of secretory material were noted (Fig. 9a, b, d). Micro-channels in cuticle occurred only in the groove cells (Fig. 5a, b). The large, prominent periplasmic space with flocculated secretory material and numerous vesicles (Figs. 5a, c, 6a–c, 7a–c, 8c, d and 9b–d) appeared in the epidermal cells of whole labellum. A noteworthy feature, observed in both hypochile and epichile, was the presence of vesicles building into the plasmalemma in the epidermis and subepidermal cells (Figs. 5a, 6a and 9a), occasionally with electron dense (possibly phenolic) substances within (Fig. 6e). The dense cytoplasm of the epidermis and subepidermal tissue contained enlarged nuclei (Figs. 5a, 8c and 9a), numerous mitochondria (occasionally in degenerative stage) (Figs. 5d, e, 7a, c, d, 8a, c, d and 9a–e), the profiles of SER and RER and fully developed dictyosomes (Figs. 5a, c, 7a–c, 8b and 9a, c–e). Lipid droplets occurred in the prickles and epichile (Figs. 7d and 9d). Additionally, an abundance of plastids, including chromoplasts with plastoglobuli (Figs. 5d, 8b and 9e) and amyloplasts with starch grains, and tubules (Figs. 5d, e, 7b, 8a–d and 9d) were present. Numerous plasmodesmata connected adjacent epidermal cells (Fig. 9e).

All these results are summarized and compared with those of previously published species from the section *Lepidorhiza* in Table 1.

**Table 1:** A comparison of macromorphology, anatomy, micromorphology, and ultrastructure of labellum of *B. levanae* and *B. nymphopolitanum* (Wiśniewska et al. 2018) and the examined *B. echinolabium* (for the need of this presentation, hypochile and epichile are presented together). + present, − absent

Species/method	*B. levanae* (Wiśniewska et al. 2018)	*B. nymphopolitanum* (Wiśniewska et al. 2018)	*B. echinolabium*
	Lip	Lip	Lip (hypochile and epichile)
Osmophores (LM: mb, methylene blue)	**+**		
Dihydroxyphenols in cytoplasm (possibly in plastids) (FeCl_3_)	**+**	**+**	**–**
Dihydroxyphenols in vacuoles (FeCl_3_)	**–**	**–**	**–**
Secretions (SEM)	**+**	**+**	**+**
Proteins (ABB)	**+**	**+**	**+**
Starch grains (PAS)	**+**	**–**	**+**
Mucilage (Ruthenium red)	**–**	**–**	**–**
Auramine O		+	+
DAPI			+
Lipids (SBB)	**+**	**+**	**+**
Secretions (TEM)	**+**	**+**	**+**
Micro-channels (TEM)	**+**	**–**	**–**
Periplasmic spaces	**+**	**+**	**+**

Living flowers emitted a strong, unpleasant smell of rotten waste. The analysis of the volatile fraction revealed the presence of a number of compounds (Table 2), mainly Diptera attractants (glycerol 1-palmitate, hexadecane, tridecane, decanal, nonanal, undecane, beta.-Linalool, limonene, 2-Hexenal, Cholest-5-en-3-ol), but also pheromones, allomones and kairomones of other insects (Hymenoptera, Heteroptera, Coleoptera, Lepidoptera) and some Carnivora. GC/MS analysis revealed that the methanol extract of lip tissue (Table 3) comprised mainly of 29.55% of different carbohydrates and their derivatives (mono- and polysaccharides, sugar alcohols, sugar acids). The main organic compound was cyanuric acid (23.61%), which is also obtained by urea decomposition.

**Table 2:** Chemical composition of labellum (hypochile and epichile together) of *Bulbophyllum echinolabium* J.J. Sm. (GC/MS analysis, extract from dichloromethane) in the context of its impact on Diptera

Dichloromethane	%	Properties and occurrence (based on El-Sayed 2019)
**Diptera attractants**		
Cholest-5-en-3-ol	5.9	Pheromone *Lucilia sericata* (Calliphoridae), Hymenoptera, Lepidoptera and Vertebrata (*i.a.* Bufonidae, Colubridae, Pythonidae, Iguanidae)
Glycerol 1-palmitate (hexadecanoic acid)	4.97	Attractant Diptera, Coleoptera, Homoptera and Astigmata; pheromone, allomone and kairomone Coleoptera, Diptera, Ixodida, Lepidoptera and some Carnivora; noted in numerous Araceae (*Sauromatum guttatum*, *Spathiphyllum cannaefolium*, *S. floribundum*) and Orchidaceae (*Bulbophyllum involutum*, *B. ipanemense*, *B. weddellii*); present in human odour (Curran et al. 2005)
Hexadecane	3.17	Anti-inflammatory, beta-oxidant and thermogenic functions; attractant Diptera (Tephritidae and Sciaridae), pheromone Hymenoptera and Coleoptera; found in many Orchidaceae (*i.a. Aerangis* spp., *Cattleya* spp., *Dendrobium* spp., *Odontoglossum* spp., *Oprhys* spp.), Cactaceae, Araceae
Tridecane	2.16	Attractant Diptera (Chloropidae and Milichiidae), pheromone and allomone Hymenoptera, Heteroptera, Coleoptera; found mainly in Araceae, Arecaceae, Orchidaceae (*Coryanthes* spp., *Cymbidium* spp., *Ophrys* spp., *Phalaenopsis* spp.), Magnoliaceae, Moraceae
Decanal	1.85	Attractant, allomone and kairomone Diptera (Muscidae, Tephritidae and Ceratopogonidae), Coleoptera, Lepidoptera; found in many Araceae, Orchidaceae (*i.a. Aerangis* spp., *Catasetum* spp., *Cirrhopetalum* spp., *Coryanthes* spp., *Dendrobium* spp., *Gongora* spp., *Stanhopea* spp.), Cactaceae, Caryophyllaceae, Rosaceae
Nonanal	1.48	Attractant Coleoptera, several Diptera (Tephritidae, Muscidae, Culicidae); found in Araceae, Arecaceae, Orchidaceae (*i.a. Aerangis* spp., *Catasetum* spp., *Coryanthes* spp., *Dendrobium* spp., *Gongora* spp., *Masdevallia* spp., *Ophrys* spp.), Cactaceae, Caryophyllaceae, Clusiaceae
Undecane	1.47	Attractant Diptera (Chloropidae, Milichiidae and Muscidae); allomone and pheromone Hymenoptera and Coleoptera found mainly in Araceae, Arecaceae, Orchidaceae (i.e. *Oprhys* spp., *Stanhopea* spp.), Rosaceae, Hypericaceae
beta-Linalool	1.31	Attractant, allomone, pheromone and kairomone Diptera (Anthomyiidae, Drosophilidae, Muscidae, Sarcophagidae, Scatopsidae, Tachinidae), Hymenoptera, Coleoptera and Lepidoptera; found mainly in Apocynaceae, Araceae, Apiaceae, Arecaceae, Brassicaceae, Orchidaceae (*i.a. Aerangis* spp., *Brassavola* spp., *Catasetum* spp., *Coryanthes* spp., *Dendrobium* spp., *Dracula* spp., *Gongora* spp., *Masdevallia* spp., *Maxillaria* spp., *Oprhys* spp., *Stanhopea* spp.), Cactaceae, Caryophyllaceae, Nyctaginaceae, Fabaceae, Magnoliaceae
Limonene	1.18	Attractant Diptera (Milichiidae, Tephritidae, Chloropidae and Dolichopodidae); Dominant compound (39%) in horse dung odour (Johnson and Jurgens 2010) occurs in other Orchidaceae (*i.a. Cattleya* spp., *Coryanthes* spp., *Dendrobium* spp., *Oprhys* spp., *Stanhopea* spp.)
2-Hexenal	0.56	Attractant Diptera (Milichiidae, Chloropidae and Tachinidae, Tephritidae, Phoridae, Psilidae); found mainly in Cactaceae, Fabaceae, Lauraceae, Cupressaceae
**Pheromones, allomones and kairomones of other insects (Coleoptera, Hymenoptera, Lepidoptera), arachnids (Astigmata) and Vertebrata (Canidae, Iguanidae)**		
Cholest-4-en-3-one	6.08	Pheromone Vertebrata (Canidae, Iguanidea)
Hexadecen-1-ol, trans-9-	4.13	Pheromone Lepidoptera and Hymenoptera (*Bombus* sp.)
Dodecanoic acid, 1-methylethyl ester	3.08	Allomone and pheromone Coleoptera, pheromone Hymenoptera
Tetradecane	2.46	Pheromone and allomone Hymenoptera, Heteroptera, Coleoptera, Astigmata
Dodecane	1.83	Pheromone and allomone Hymenoptera, Coleoptera; found in Araceae and Orchidaceae
**Properties and occurrence of other constituents (in context of fly pollination or medical significance)**		
Tridecanol, 2-ethyl-2-methyl-	5.56	Noted in *Acalypha wilkesiana* (Euphorbiaceae) leaves, may be useful in the management of risk factors of cardiovascular diseases (Omage et al. 2018)
Benzenepropanoic acid, 3,5-bis(1,1-dimethylethyl)-4-hydroxy-, methyl ester (Metilox)	3.83	According to OECD (2001), SIAM 13, 6-9.11.2001 this chemical has no concern for health and for environment, currently of low priority for further work
7,9-Di-tert-butyl-1-oxaspiro(4,5)deca-6,9-diene-2,8-dione	3.79	Found in essential oils from *Cordia sebestena* (Boraginaceae) (Adeosun et al. 2013)
Isopropyl 12-methyl-tridecanoate	3.56	–
2,3,5,6-Detetrahydrocyclohexanone, 2,6-di-t-butyl-4-hydroxymethylene-	3.43	–
Octylacetophenone	3.09	–
Heptadecane, 4-methyl-	2.91	–
Benzoic acid, 3-ethyloxy-, ethyl ester	2.81	–
Phenol, 3,5-di-tert-butyl-	2.78	Phenol derivative, found in essential oils of *Azadirachta indica*, *Azadirachta siamensis* and *Azadirachta excelsa* (Meliaceae) (Kurose and Yatagai 2005)
2,5-di-tert-Butyl-1,4-benzoquinone	2.65	Neurotoxic for humans (O'Donoghue 1985), antimicrobial activity against *Bacillus cereus* (Gopal et al. 2013)
Butoxyethoxyethyl acetate (2-(2-Butoxyethoxy)ethyl acetate)	2.36	May cause an allergic skin reaction and respiratory irritation in vertebrates (European Chemicals Agency 2019)
Decane, 2,3,5,8-tetramethyl-	2.14	–
Dodecane, 2,6,10-trimethyl-	2.12	–
Dodecane, 2,6,11-trimethyl-	2.1	–
2-Octene, 3,7-dimethyl-	1.71	Can be found in faeces (Wishart et al. 2018)
Undecane, 4-methyl-	1.53	–
2,6-Dimethyldecane	1.48	–
1-Heptanol, 2-propyl-	1.45	–
Santolina triene	1.42	–
2-Hexanol, 1-mercapto	1.15	–
3-Octen-5-yne, 2,7-dimethyl-	1.13	–
Cyclohexane, 1-methyl-3-propyl-	0.84	–
1-Butanol, 2-ethyl-	0.76	–
Cyclohexane, 1-methyl-3-propyl-	0.74	–
3-Ethyl-2-methyl-1-heptene	0.72	Noted in ox carcass (Gikonyo et al. 2002)

**Table 3:** Chemical composition of labellum (hypochile and epichile together) of *Bulbophyllum echinolabium* J.J. Sm. (extract from methanol)

Methanol	%	Properties (based on El-Sayed 2019)
**Amino acids**		
L-phenylalanine	0.74	Amino acid
Serine	0.46	Amino acid; pheromone Hymenoptera
Norleucine	0.26	Amino acid
L-threonine	0.20	Amino acid
**Saccharides**		
alpha-D-Glucopyranoside	11.70	Monosaccharide
D-Turanose	3.84	Disaccharide
Sucrose	3.27	Disaccharide
D-Glucose	3.08	Monosaccharide
Hydroquinone-beta-d-glucopyranoside	1.53	Monosaccharide
D-Galactose	1.10	Monosaccharide
Glucopyranose	1.10	Monosaccharide
2,3,4-Trihydroxybutyric acid	1.00	Sugar acid
Glycoside, alpha-methyl	0.70	Monosaccharide
Ribonic acid	0.43	Sugar acid; can primarily be found in faeces
D-Xylopyranose	0.27	Monosaccharide
D-(−)-Tagatofuranose	0.26	Natural sweetener present in fruits, cacao and dairy products
Inositol	0.17	Polyol (multiple/sugar alcohol)
D-Ribofuranose	0.16	Monosaccharide
Erythritol	0.16	Sugar alcohol (aliphatic)
D-(+)-Xylose	0.13	Monosaccharide
Myo-Inositol	0.13	Polyol (multiple/sugar alcohol)
Fructose	0.12	Monosaccharide
beta-l-Galactopyranoside, methyl 6-deoxy-	0.09	Monosaccharide
D-Gluconic acid	0.08	Carboxylic acid formed by the oxidation of the first carbon of glucose
Levoglucosan	0.08	Anhydrohexose
d-Mannose	0.07	Aldohexose
2-Keto-d-gluconic acid	0.05	Carrageenan polysaccharide
Ribitol	0.03	Sugar alcohol
**Lipids**		
Stigmasterol	5.27	Phytosterol (lipid)
Campesterol	1.39	Phytosterol (lipid)
Cholesterol	1.22	Sterol (lipid)—attractant of blow fly *Lucillia sericata*
1-Monopalmitin	0.16	Monoacylglycerols—minor component of olive oil and other vegetable oils
**Others**		
Cyanuric acid	23.61	Obtained by urea decomposition, which is final product in the metabolism of nitrogen-containing (mainly amino acids) compounds by animals
Malic acid	22.22	Dicarboxylic acid, responsible for the pleasantly sour taste of fruits
1-Cyclohexene-1-carboxylic acid, 3,4,5-hydroxy	11.76	Intermediate metabolite in plants and microorganism
Citric acid	1.20	–
alpha-Hydroxyglutaric acid	0.59	–
Mannonic acid, 1,4-lactone	0.50	–
Benzoic acid, 3-methoxy	0.42	Flavouring ingredient for food; found in blood and urine
beta-Hydroxy-beta-methylglutaric acid	0.18	Monocarboxylic β-hydroxy acid metabolized from leucine
p-Hydroxybenzoic acid	0.17	Allomone and pheromone of Coleoptera and Hymenoptera
Pantothenic acid	0.05	Amide between pantoic acid and β-alanine
alpha-Aminoadipic acid	0.03	Intermediate in the metabolism of lysine and saccharopine (protein precursor)

## Discussion

Secretory activity was observed on the hypochile (on the longitudinal groove, keel and on the prickles) and the epichile of *Bulbophyllum echinolabium*. Considering the presence of carbohydrates (29.55% in GC/MS), the profusion of starch grains (PAS) and a large amount of the secretory material on hypochile (TEM), we consider it nectar. Nectar is mainly composed of monosaccharides (glucose and fructose) and a disaccharide (sucrose), with different concentrations among species (Percival 1965). The proportion of monosaccharides over disaccharides confirms that this hexose-rich nectar is offered for flies and was also identified in flowers pollinated by short-tongued bees, bats and perching birds (Baker and Baker 1983, 1990; Lammers and Freeman 1986; Elisens and Freeman 1988; Stiles and Freeman 1993; Baker et al. 1998; apud Perret et al. 2001). Whereas, the epichile, with cells histochemically and ultrastructurally the same as the hypochile, but with slight accumulation of exuded material on the surface, functions as a putative osmophore.

The typical histological organization, in both the hypochile and epichile of the species here studied, was distinguished as a single-layered epidermis with several layers of subepidermal parenchyma and mesophyll-like ground parenchyma with collateral vascular bundles. Such regions were previously described in species from section *Lepidorhiza* (Wiśniewska et al. 2018), Neotropical sections *Didactyle* (Nunes et al. 2014), *Napelli* (Nunes et al. 2015), *Racemosae* (Davies and Stpiczyńska 2014; Stpiczyńska and Davies 2016), African (Teixeira et al. 2004; Stpiczyńska et al. 2015) and Asian representatives of the genus (Kowalkowska et al. 2015a, 2017). Alike in other *Lepidorhiza* species, in *B. echinolabium*, the exudate could pass diffusely through the cuticle or accumulate under the cuticle and, as a result of increasing pressure, could emerge outside the cells through cuticular cracks. The prominent periplasmic space appeared in the epidermal cells of both the hypochile and epichile. Interestingly, subcuticular spaces were present in two other species from the section *Lepidorhiza* (Wiśniewska et al. 2018). The formation of periplasmic spaces is probably connected with merocrine secretion, where glands or cells remain alive after secretion and continue their secretory activity. Generally, our observations of *B. echinolabium* support the hypothesis proposed by Paiva (2016), where synthesized substances are transported via vesicles to the periplasmic space (granulocrine secretion), from which they are transported out through the cuticle as a result of increasing pressure. The presence of the irregular plasmalemma, secretory vesicles fusing with it and fully developed dictyosomes also indicates granulocrine secretion, as reported previously in orchids (i.e. Kowalkowska et al. 2012, 2015b; Wiśniewska et al. 2018). Moreover, the enlarged nucleus, as well as the abundance of mitochondria, indicate high cellular activity. In the labellum of *B. echinolabium*, a large number of idioblasts contained raphides of calcium oxalate crystals, which play a significant role in deterring herbivores. Idioblasts located close to the secretory tissue in tepals are a common feature and are frequently noted (i.e. Kowalkowska et al. 2015b; Wiśniewska et al. 2018).

A great number of compounds identified in the whole lip are Diptera attractants. Fifteen out of 43 volatile compounds found in *B. echinolabium* are described in the Pherobase (El-Sayed 2019) as semiochemicals (attractants, pheromones, allomones or kairomones). Most of them specifically attract Diptera (mostly Milichiidae, Muscidae, Drosophilidae, Sarcophagidae, Chloropidae, Tephritidae and Tachinidae). Semiochemicals are natural chemicals released by organisms and used as a main channel of communication. The signals, intraspecific or interspecific, are used in many behavioural functions, including sexual attraction, aggregation, trail following, recruitment, defence or host location (El-Sayed 2019). Furthermore, two other semiochemicals might also attract flies floral odours—3-ethyl-2-methyl-1-heptene and 3,7-dimethyl-2-octene are also detected in the faeces and the body odour composition of ox, respectively (Gikonyo et al. 2002; Wishart et al. 2018). Limonene, in turn, was noted in small amounts in *Ferraria crispa* (Johnson and Jürgens 2010), as well as in horse dung, where it was a dominant compound (39%). Terpinen-4-ol occurs in many species of Orchidaceae (263 species described in the Pherobase) and likewise in two fly-pollinated species from Araceae: *Arum maculatum* and *Sauromatum guttatum* (El-Sayed 2019).

Profusion of proteins present in labellar secretion, also noted in *B. nymphopolitanum* and *B. levanae* (Wiśniewska et al. 2018), is probably connected with the unpleasant scent of the flowers. During active decay in carcasses, protein sources are broken down into nitrogen, phosphorus and sulphur compounds (Statheropoulos et al. 2005). Cyanuric acid, which is the final product in the metabolism of nitrogen-containing (urea decomposition) compounds by animals (Kim et al. 2019), accounted for almost a quarter of all compounds. Likewise, sugar and sugar alcohols may act as insect pheromones or semiochemicals, as in the case of apple fruit surfaces, where they have been demonstrated to function as kairomones and stimulate egg laying in female moths of *Cydia pomonella* L. (Lombarkia and Derridj 2002). Furthermore, the phenolic derivatives present in the floral scent of *B. echinolabium* are known to occur in the floral scent of sapromyophilous species, e.g. *Caralluma europea* (Formisano et al. 2009), *Duvalia corderoyi*, *Hoodia gordonii*, *Orbea variegata* (Jürgens et al. 2006), *Stapelia leendertziae*, *O. verrucosa*, *Euphorbia grandicorni*s (Johnson and Jürgens 2010), as well as in several types of decaying organic matter, such as dog (Arnould et al. 1998), rabbit (Goodrich et al. 1981) and cow faeces (Kite 1995). The electron dense secretion residues on and beneath the cuticle surface and in plastoglobuli in plastids are possibly phenolic in nature. In *B. echinolabium*, ER profiles were closely associated with plastids containing plastoglobuli, as well as numerous vesicles with electron dense material, fusing with plasmalemma. The volatile (phenolic) components of fragrances are synthesized in plastoglobuli: hence, they are transported to the intraplastidal membranes, crossing the plastid envelope to the profiles of ER (or migrating independently in cytoplasm) and finally to the plasmalemma, where they exit the cells. Plastid involvement in the synthesis of fragrance components has been described previously (Stern et al. 1987; Kowalkowska et al. 2012; Stpiczyńska and Davies 2016) and in other *Lepidorhiza* representatives (Wiśniewska et al. 2018).

Cuticular striations covered with epicuticular wax layers (stained with Auramine O) present on the *B. echinolabium* labellum adaxial surface could cause the brilliance of floral tepals as well as shining nectar. Meve and Liede (1994) called it “mimetic surface reflexion”, where glistering surfaces strongly attract flies by imitating open flesh wounds or fresh dung surfaces. Furthermore, residues might function as a visitor guide to align the mouth parts of the flies to achieve successful pollinaria transfer (Endress 1994; Meve and Liede 1994; Jürgens et al. 2006). Carnivorous plants from the genus *Nepenthes* are covered with a thick layer of epicuticular wax, forming slippery zones for pollinators, which seem to play a crucial role in animal trapping. Tepal striations are considered to intensify floral attraction for pollinators (in *Bulbophyllum*—Nunes et al. 2015). Furthermore, substances contained in epicuticular waxes function as allomones, deterring oviposition and feeding by herbivores (Eigenbrode and Espelie 1995; Müller and Riederer 2005).

In conclusion, our studies indicate that, despite differences in size and colour, *B. echinolabium* and previously studied *B. levanae* and *B. nymphopolitanum* have many similarities. The unusual cell wall structure seems to be a common feature among the section *Lepidorhiza* and sheds more light on secretion in sapromyophilous Orchidaceae. The odour composition seems to mimic the odour of carcasses described previously in stapeliads (Jürgens et al. 2006; Płachno et al. 2010). Furthermore, the reddish and purplish colour together with the echinate texture of the labellum reinforce the scent mimicry. The importance of both floral visual and olfactory features in attracting flies is explained by the experimental data carried out by Beaman et al. (1988). After removing visual cues, blue bottle flies were present on the flowers of *Rafflesia pricei* only 35% of the time. Likewise, when fragrance was removed, flies were present only 7–47% of the time (depending on the effectiveness of fragrance removal). When both olfactory and visual attractants were present, blue bottle flies were noted on the flowers for 95% of the observation time. It is of interest whether correlation of olfactory and visual effects are common in different sapromyophilous species. For Orchidaceae, such analyses of fly-pollinated species are still lacking.
